# Thematic orders and the comprehension of subject-extracted relative clauses in Mandarin Chinese

**DOI:** 10.3389/fpsyg.2015.01255

**Published:** 2015-09-11

**Authors:** Chien-Jer Charles Lin

**Affiliations:** Department of East Asian Languages and Cultures, Indiana UniversityBloomington, IN, USA

**Keywords:** sentence comprehension, thematic orders, relative clauses, expectations, Mandarin Chinese

## Abstract

This study investigates the comprehension of three kinds of subject-extracted relative clauses (SRs) in Mandarin Chinese: standard SRs, relative clauses involving the disposal *ba* construction (“disposal SRs”), and relative clauses involving the long passive *bei* constructions (“passive SRs”). In a self-paced reading experiment, the regions before the relativizer (where the sentential fragments are temporarily ambiguous) showed reading patterns consistent with expectation-based incremental processing: standard SRs, with the highest constructional frequency and the least complex syntactic structure, were processed faster than the other two variants. However, in the regions after the relativizer and the head noun where the existence of a relative clause is unambiguously indicated, a top-down global effect of thematic ordering was observed: passive SRs, whose thematic role order conforms to the canonical thematic order of Chinese, were read faster than both the standard SRs and the disposal SRs. Taken together, these results suggest that two expectation-based processing factors are involved in the comprehension of Chinese relative clauses, including both the structural probabilities of pre-relativizer constituents and the overall surface thematic orders in the relative clauses.

## Introduction

Relative clauses have been of great theoretical interest to sentence processing researchers, with decades of research comparing the processing of subject-extracted relative clauses (henceforth “SRs”) to that of object-extracted relative clauses (henceforth “ORs”). A robust asymmetry has been repeatedly reported in languages where the relative clauses follow the nouns they modify (i.e., languages with head-initial relative clauses). In English, for example, relative clauses involving subject extractions like (1) have been found to be easier to comprehend than those involving object extractions like (2) (Ford, 1983; King and Just, 1991; King and Kutas, 1995; Gibson et al., 2005; Traxler et al., 2005). The head noun phrases in these constructions [indicated with boldface in (1, 2)] are conventionally referred to as the *fillers* in the sense that they fill the *gaps* located at the extracted positions in the subordinate clauses [indicated with underscores in (1, 2)].

(1) Subject-extracted relative clause:
       {**The composer**_i_ who ___i_ adored the musician} drank a glass of wine.
(2) Object-extracted relative clause:
       {**The musician**_i_ who the composer adored ___i_} drank a glass of wine.

Two main groups of theories have been adopted to account for this processing asymmetry, here referred to as “integration-based theories” and “experience-based theories.” The first group of theories focuses on the consumption of working memory in constructing filler-gap dependencies, suggesting that SRs in English are easier to comprehend (with shorter reading times and greater comprehension accuracies) because, relative to ORs, less working memory is required to process them. Within these integration-based theories, a number of proposals have been made as to precisely how the relevant processing costs are computed. A linearity account (e.g., Gibson, 1998) focuses on the number of referents intervening between the filler and the gap, attributing the easier comprehension of SRs to fewer new referents intervening between the filler and the gap. As a filler is assumed to remain active until a gap is reached in constructing filler-gap dependencies, the longer filler-gap distance in an OR consumes greater processing costs. A relevant variant of the linearity account focuses on the types of noun phrases intervening the filler and the gap, according to which similar types of noun phrases (NPs) create greater interference and therefore induce higher processing costs (Gordon et al., 2001). The activation and cue-based retrieval theory (Van Dyke and Lewis, 2003; Lewis and Vasishth, 2005) on the other hand takes into consideration the lexical items intervening a filler and a gap as contributors to activation and retrieval.

**Figure 1:**
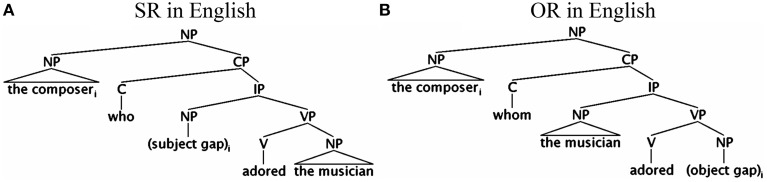
**Syntactic structure of relative clauses in English**.

Within the integration-based theories, a structural account (e.g., O'Grady, 1997; Miyamoto and Nakamura, 2003; Hawkins, 2004; Lin, 2006) relies on the structural distance between the filler and the gap (e.g., computed by counting the number of intervening XPs) to compute processing costs. On this account, processing costs are determined by the number of intervening structural nodes inside a filler-gap dependency. Thus, an SR is easier to comprehend than an OR in English because a subject gap is structurally higher and closer to the relative clause operator (i.e., the complementizer *who/whom*) than an object gap (see Figure 1). Since fewer structural nodes intervene between the operator and the subject gap, less working memory is consumed in connecting the filler with the subject gap^1^.

The second group of theories is experience-based, formalized either as constraints (e.g., MacDonald and Christiansen, 2002; Raeli and Christiansen, 2007; Gennari and MacDonald, 2008) or through a construct of “expectation” (Surprisal: Hale, 2001; Levy, 2008; Entropy Reduction: Hale, 2006). These theories account for the processing differences by resorting to probabilistic information associated with one's linguistic experiences, attributing the easier processing of SRs to the greater structural predictability associated with SRs than ORs. Since SRs have a higher frequency of occurrence than ORs in English (Roland et al., 2007), the parser is more likely to parse the head noun and relativizer in English as starting an SR than an OR. Thus, the increased predictability associated with SRs is claimed to be what induces the shorter reading times.

A related experience-based theory posits that the dominant (i.e., most frequent) thematic order in a language can be used as a perceptual strategy to facilitate sentence comprehension (Bever, 1970; Townsend and Bever, 2001; Lin, 2013). According to the thematic-order account, experience with thematic orders form *canonical thematic templates*, which may facilitate efficient thematic interpretations. Since the canonical word order in English is SVO and the canonical thematic order is agent-verb-patient, SRs, which present orders consistent with the dominant order, are predicted to be less costly to process^2^. The thematic order account predicts increases in reading time where word order mismatches take place.

This brief summary highlights the fact that the overall advantageous reading of SRs in English is consistent with multiple theories of sentence comprehension though specific predictions about where the processing differences should be observed may differ. Gibson and Wu (2013), for example, point out that an integration account predicts the increase in processing load where the filler-gap integration takes place (i.e., around the embedded verb region in head-initial relative clauses). An experience-based account, on the other hand, predicts the increase in processing load should occur where processing uncertainty increases (i.e., around the embedded subject but not on the embedded verb in an OR).

An accumulating body of research over the past decade has painted a somewhat different picture of the processing of head-final relative clauses (Basque: Carreiras et al., 2010; Japanese: Miyamoto and Nakamura, 2003; Ueno and Garnsey, 2008; Korean: Kwon et al., 2010; Mandarin Chinese: Hsiao and Gibson, 2003; Lin and Bever, 2006; Lin and Garnsey, 2011; Packard et al., 2011; Qiao et al., 2012; Gibson and Wu, 2013; Jäger et al., 2015; Turkish: Kahraman et al., 2010). By definition, in a head-final relative clause construction, the relative clause appears before the head noun it modifies, meaning that the gap is encountered before the filler (rather than after it, as in English). Such structures are of crucial theoretical interest since they make it possible to reexamine the predictive power of the different competing sentence comprehension theories in a new context.

To illustrate the theoretical relevance of head-final relative clause processing, consider the Mandarin Chinese (henceforth “Chinese”) sentences with relative clauses in (3) and (4)^3^.

(3) Sentence with subject-extracted relative clause in Chinese:
        __i_ aimu  yinyuejia  de    zuoqujia_i_    he-le         yi   bei     jiu
        __i_ adore musician  rel composer_i_ drink-asp  one glass wine
        “The composer who adored the musician drank a glass of wine.”
(4) Sentence with object-extracted relative clause in Chinese:
        zuoqujia    aimu  __i_ de   yinyuejia_i_ he-le         yi   bei     jiu
        composer adore __i_ rel musician_i_ drink-asp one glass wine
        “The musician whom the composer adored drank a glass of wine.”

**Figure 2:**
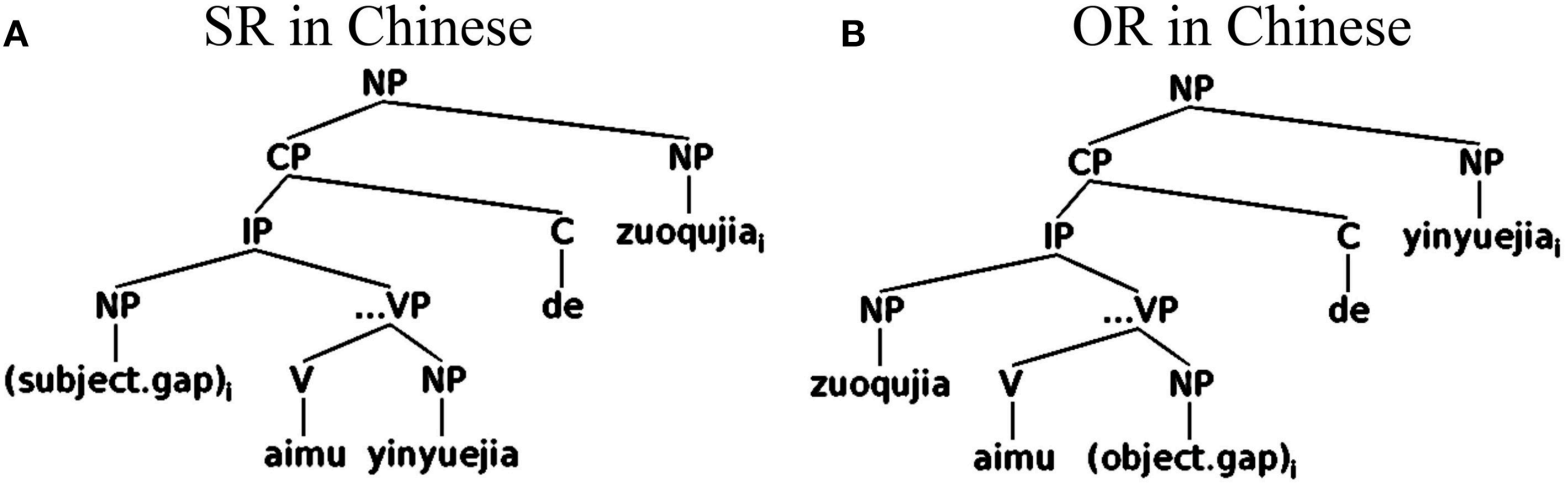
**Syntactic structure of relative clauses in Chinese**.

Chinese displays a head-initial structure in verb phrases: like English, Chinese is a Subject-Verb-Object (SVO) language, with verbs preceding their NP object complements. At the same time, however, Chinese displays a head-final structure in NP: modifiers of nouns exclusively appear before the head noun. Because of this combination, while subject gaps in Chinese are higher and structurally closer to the complementizer/relativizer than object gaps (as in English), subject gaps are linearly *farther* from the head noun (i.e., the filler) than object gaps, unlike English. These facts are illustrated in Figure 2, which diagrams the relative clauses from (3, 4)^4^.

Regarding gap-filler integration, therefore, the linearity account predicts that the gap-filler relation in a Chinese OR should be less taxing to construct than that in an SR. The structure-based account predicts the opposite: since fewer structural nodes intervene between the head noun and a subject gap, the dependency between these two should be easier to construct compared to one involving an object gap. Both accounts would predict the locus of processing differences on the head noun where gap-filler integrations take place.

Regarding the effect of structural probabilities, given that SRs have higher frequencies than ORs in Chinese (Wu et al., 2011), greater surprisal values are associated with ORs and thus longer reading times in ORs are predicted. In terms of the effect of dominant thematic orders, since the canonical thematic order in Chinese is agent-verb-patient, ORs, which follow the dominant order, are predicted to be less costly to process (Lin, 2013, 2014). The experience-based effects make processing predictions for the whole sentences based on structural and word-order probabilities, not just for particular regions where integration costs incur.

Chinese has thus been taken as a valuable test case for validating the integration-based accounts and experience-based accounts depicted above (see also Jäger et al., 2015 for a review of the theoretical controversy). So far, research has provided a somewhat mixed picture. In the head-noun region, some studies have found that ORs took longer to read than SRs (Lin and Bever, 2006; Chen et al., 2012; Jäger et al., 2015) while others found the opposite (Gibson and Wu, 2013). One potential difficulty in acquiring consistent results is that studies differed regarding whether and how relative clauses are motivated. When relative clauses are not motivated (for example, when they appear in isolated sentences without referential contexts or structural cues preceding them), surprisal effects related to reanalyses may induce longer reading times in the disambiguating regions. Gibson and Wu (2013), for instance, pointed out that Chinese ORs may be more difficult to comprehend than SRs in neutral contexts because ORs are more likely to induce a garden path effect in the prenominal regions. Longer reading times for ORs are thus predicted in the head noun region, where disambiguation takes place.

On the other hand, when relative clauses are pragmatically motivated or structurally disambiguated, one needs to consider the potential effects of the different contextual cues. Chinese relative clauses have previously been pragmatically motivated by using discourse contexts (Gibson and Wu, 2013; Lin, 2014), and structurally disambiguated by using classifier-noun mismatching cues (Hsu et al., 2006) and classifier-adverbial sequences (Jäger et al., 2015). In studies that motivated relative clauses by using referential contexts (Lin, 2014; cf. Gibson and Wu, 2013), relative clause processing was shown to be sensitive to the order of thematic roles in the context: relative clauses whose thematic orders match those in the referential contexts showed shorter reading times in the regions after the head noun. In studies where relative clauses were structurally disambiguated, reading patterns have been found to be consistent with the conditional structural probabilities of SRs and ORs. Jäger et al. (2015), for instance, reported reading patterns consistent with surprisal predictions based on a corpus study and a sentence completion task. In Chinese relative clauses that follow disambiguating syntactic contexts like classifier-adverbial sequences, the conditional probability of an SR is higher than that of an OR in the embedded clause regions (i.e., IPs in Figure 2) but not on the head noun. Reading patterns confirmed that an SR advantage existed in the embedded clause regions but not on the head noun.

Methodologically, processing studies comparing Chinese SRs and ORs have reached a bottleneck. In most previous studies, SRs have been directly compared to ORs, meaning that SRs and ORs serve as each other's baseline conditions. Accordingly, any processing difference between the two has typically been associated with one single factor of theoretical interest. For instance, Gibson (1998) focuses on differences in linear distance between the gap and the filler, whereas theories of structural complexity (O'Grady, 1997; Hawkins, 2004) focus on differences in the number of structural layers/nodes intervening between the two. In fact, however, SRs and ORs are different from each other in multiple ways beyond these differences. In terms of constructional frequencies, SRs are more common than ORs (Lin, 2009; Wu et al., 2011). In terms of structural predictability, an SR is better expected than an OR (Jäger et al., 2015). In terms of nominal animacy preferences, the heads of SRs are preferably animate while those of ORs are preferably inanimate (Wu et al., 2012). Because SRs and ORs are simultaneously different from each other in so many ways, results from previous studies comparing the two are difficult to interpret.

The present study addresses this methodological issue by holding the extraction site constant (only SRs) and investigating the processing of three different *sub-types* of SRs: standard SRs, SRs with the disposal *ba* construction (henceforth “disposal SRs”), and SRs with the long passive *bei* construction (henceforth “passive SRs”). Both the disposal *ba* construction and the passive *bei* construction involve functional morphemes that have been analyzed as light verbs or grammaticalized verbs in Mandarin Chinese. An example for each construction is given in (5–7). Sentences with relative clauses appear after referential contexts, which are intended to pragmatically motivate relative clauses so that the prenominal relative clauses are parsed as relative clauses when they appear in sentences.

(5) Standard SR:
        ___i_ jiaoxing  furen de   zuoqujia_i_    he     yi    bei     jiu
        ___i_ wake.up lady   rel composer_i_ drink one glass wine
        *      action   patient     agent*
        “The composer that woke up the lady drank a glass of wine.”
(6) Long passive (*bei*) SR:
        ___i_ bei  furen   jiaoxing  de    zuoqujia_i_    he     yi    bei    jiu
        ___i_ BEI lady    wake.up rel  composer_i_ drink one glass wine
                    *agent action            patient*
        “The composer that was woken up by the lady drank a glass of wine.”
(7) Disposal (*ba*) SR:
        ___i_ ba   furen     jiaoxing de   zuoqujia_i_    he      yi   bei
        ___i_ BA lady      wake.up rel composer_i_ drink one glass
                   *patient action           agent*
              jiu
             wine
        “The composer that woke up the lady drank a glass of wine.”

Being SRs, all three structures involve the extraction and relativization of the subject NP, which, in Chinese, involves a movement type of dependency between the subject gap and the head NP (Aoun and Li, 2003). Where these three structures differ from one another is the internal structure of the pre-relativizer inflectional phrase (IP)—in particular, the structure of the verb phrase (VP) and the small verb phrase (vP) following the subject gap. Each of these three constructions will now be discussed in turn.

**Figure 3:**
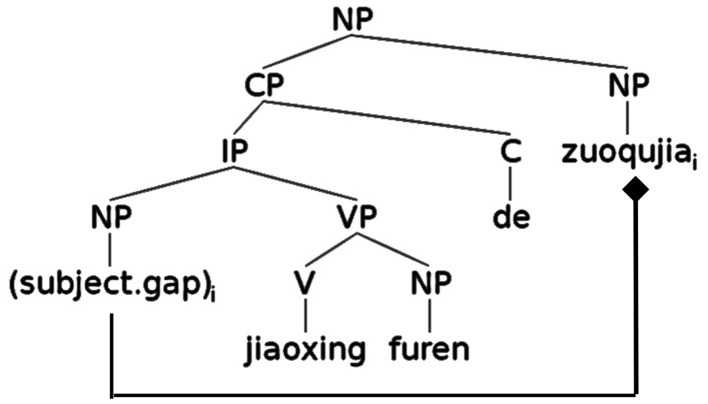
**Syntactic structure of a standard SR in Chinese**.

The syntactic structure of a standard SR is illustrated in Figure 3, representing the relative clause portion of (5). Standard SRs contain an SVO sequence with an empty subject NP inside the IP.

**Figure 4:**
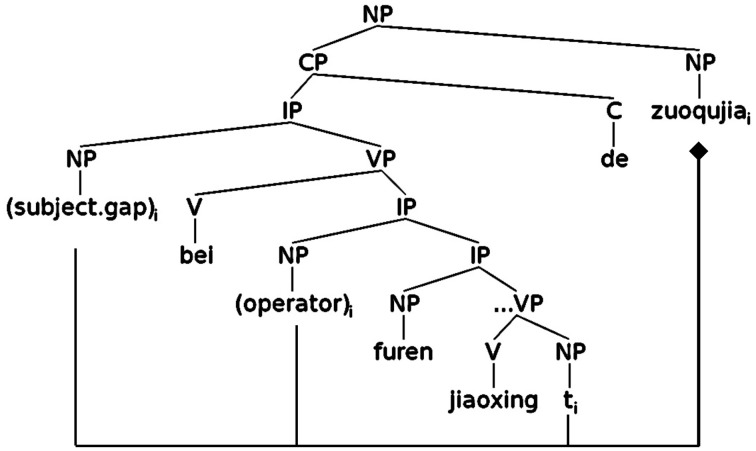
**Syntactic structure of a passive SR in Chinese**.

The syntactic structure of a passive SR is illustrated in Figure 4, representing the relative clause portion of (6) above. Under the main-verb analysis for Chinese long passives (Huang et al., 2009), this structure contains an empty subject and a VP headed by *bei*, followed by a secondary predicate IP^5^. Three dependencies are involved in this construction. First, as in all the SRs, a dependency exists between the subject gap and the head NP. Second, an additional dependency exists between the base generated object NP position in the lower VP and the NP operator at the periphery of the intermediate IP. Third, this NP operator holds the same identity as the subject gap. The three empty positions (the subject gap, the operator, and the trace) all bear the same identity as the head NP.

**Figure 5:**
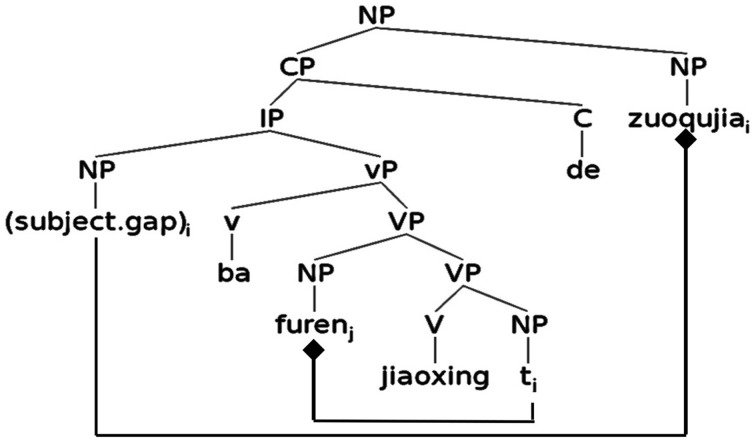
**Syntactic structure of a disposal SR in Chinese**.

The structure of a disposal SR is illustrated in Figure 5, representing the relative clause portion of (7) above. Like passive SRs, disposal SRs also involve multiple dependencies. Under the light verb analysis of *ba* (e.g., Huang, 1997; Lin, 2001), the object NP of the lower VP is displaced to the specifier position. Two separate dependencies involving empty categories need to be constructed in the processing of a disposal SR: one between the subject gap and the head NP (outer connection in Figure 5), and the other between the moved object NP immediately following *ba* and the position of its trace (inner connection in Figure 5). Unlike passive SRs, the VP-internal dependency in a disposal SR is nested inside the dependency between the subject gap and the head NP^6^.

The processing factors discussed above cast different effects on these three types of Chinese SRs. Let's first focus on the integration effects regarding the dependency between the subject gap and the head noun in each of the three structures, which are usually taken to be observable around the head noun region, where filler-gap integrations take place. In terms of linear locality (Gibson, 1998; Hsiao and Gibson, 2003), the same numbers of *new referents* intervene between the gap and the filler, thus predicting no processing differences. If linear distance is computed using the number of intervening *words*, then the passive SRs and the disposal SRs may both require greater processing load than the standard SRs because they involve an additional function word (*bei* and *ba*, respectively) between the gap and the filler. In terms of structural locality, since all three SRs involve extraction from subject position, the structural distance between the head noun and the gap are identical across all three structures (passing through two XP nodes—one CP and one IP), thus predicting no processing differences.

In addition to the gap-filler dependencies, the passive SRs and the disposal SRs involve additional displacement dependencies as depicted in Figures 4, 5. For a passive SR, the sentence-initial passive marker *bei* indicates a missing subject NP that is to be connected with the object NP in the lower VP. Assuming that a relative clause parse has been adopted, the missing subject NP is taken to be connected both with the object NP and the head noun^7^. For a disposal SR, the sentence-initial light verb *ba* also indicates a missing subject NP. Assuming again the processing of a relative clause, this missing subject NP would be taken as a subject gap connected with the head noun. An additional dependency in a disposal SR involving the displaced object NP after *ba* would add to the processing cost already incurred by the SR. The integration-based accounts, taking into consideration these additional dependencies, would then predict that both a passive SR and a disposal SR should be harder than a standard SR because (a) the former SRs involve additional dependencies, and (b) the dependencies in the former SRs are longer and more complex than that of a standard SR. Processing differences are expected to appear on and before the head noun.

Next, we consider the overall structural complexity and structural frequencies involved in the three types of SRs. The standard SR is the simplest of the three constructions, as it contains the fewest number of structural layers and only has a single dependency relation (between the subject gap and the head). Passive SRs and disposal SRs are both more complicated in terms of the intricate dependency relations inside the VP/vP^8^. This hierarchy of complexity is consistent with the constructional frequencies of the 3075 relative clauses extracted from the Sinica Treebank (Version 3.0; Chen et al., 2003) by Lin (2009), among which standard SRs accounted for 53%, passive SRs accounted for 2%, and no instances of disposal SRs were found^9^. Thus, based on both structural complexity and constructional frequency, a standard SR should be the easiest to process among the three.

On the other hand, the thematic order effect predicts different processing preferences. Since the surface thematic order of a passive SR matches the canonical thematic order in Chinese (i.e., agent-*action*-patient), a passive SR should be the easiest to process among the three. Conversely, the thematic orders of standard SRs and disposal SRs are inconsistent with this dominant thematic order and should be more difficult to process than the passive relatives.

One relevant hypothesis about effect locus proposed by Lin (2014) is that the pre-relativizer and post-relativizer regions of a head-final relative clause may reveal different processing effects. This hypothesis is directly related to the existence of uncertainty in processing head-final relative clauses: while the pre-relativizer regions are structurally ambiguous, the post-relativizer regions are structurally unambiguous. The pre-relativizer regions of an OR, for example, with the word order *Noun-Verb*, are more likely to be read as matrix clauses than subordinate relative clauses. The corresponding pre-relativizer *Verb-Noun* sequence of an SR would be parsed as a matrix clause with a missing subject argument before the verb (see Lin and Bever, 2011; Jäger et al., 2015 for more elaborated discussion on the issue of garden path in Chinese relative clauses). With the post-relativizer regions, however, no similar ambiguity exists since comprehenders tend to parse the functional morpheme *de* after the embedded clause as a relativizer. A corpus study and a sentence completion task by Jäger et al. (2015) have confirmed that a relative clause parse is already unambiguously established when the relativizer is reached. Lin (2014), in particular, proposes the effect of thematic templates, being a pattern matching effect, may be more observable in the post-relativizer regions where structural uncertainty has decreased.

In addition to the overall predictions of the effects, we thus further distinguish the processing effects in the pre-relativizer regions and the post-relativizer regions. In the pre-relativizer regions, disposal SRs and passive SRs are both expected to take longer to process than standard SRs given greater structural complexity and lower structural frequencies^10^. Integration effects based on linear locality and structural locality would make similar predictions given that simpler dependent relations exist in the standard SRs than in the disposal and passive SRs. The effect of thematic template mapping, on the other hand, predicts shorter reading time for passive SRs because they display thematic orders consistent with the canonical order in Chinese though this effect may emerge later in a prenominal relative clause construction.

In the post-relativizer regions, where the existence of relative clauses are clearly indicated by the relativizers and the head nouns, an integration account based on linear locality would predict that standard SRs be easier than both disposal and passive SRs, especially around the head noun region. An integration account based on structural locality would predict no processing differences, or easier processing for standard SRs due to the complexity effect possibly spilled over from the prenominal regions. The effect of thematic template mapping is the only theory that predicts an overturned reading pattern for passive SRs, with passive SRs being the least costly to read. The effect of thematic template mapping is expected to span across multiple post-relativizer regions.

The goal of the present study, in summary, is to examine the effect of thematic orders on Chinese relative clause processing. While Lin (2014) reported that the processing of SRs and ORs in Chinese is sensitive to the thematic orders presented in the context, it directly compared the processing of SRs and ORs, which as discussed, involve an array of differences that may obscure the effects. The present study contrasted the processing of three sub-types of SRs, thus keeping constant the extraction site regarding its grammatical function in the embedded clause. Furthermore, Lin (2014) studied the effect of thematic orders by varying the orders in the referential context while keeping the thematic orders in the relative clauses constant. The present study examined this effect by varying the thematic orders in the relative clauses while keeping the thematic orders in the context constant. It is hoped that this new manipulation can test the effect of thematic order on relative clause processing from a new angle.

## Materials and methods

### Participants

Forty-eight Taiwanese college students at National Taiwan Normal University, all native speakers of Mandarin Chinese, participated in the experiment. The participants were screened for brain damage. All had normal (or corrected to normal) vision, and were naïve to the purpose of the experiment. Participants gave informed consent to take part in the study. The study protocol was approved by Indiana University's Institutional Review Board.

### Materials

Twenty sets of sentences were included as the experimental trial, 16 of which were modified based on Gibson and Wu's (2013) stimuli. The experimental materials were created in such a way that they read naturally in Mandarin disposal and passive constructions. To motivate the relative clauses, each set consisted of a referential context introducing transitive relations in which three referents are involved, as in (8). The sentences in the context where these thematic relations were introduced present the thematic order of agent-*action*-patient. Following each context was a dialogue between two interlocutors, Xiaoming and Xiaomei, in which Xiaoming asks Xiaomei to identify one referent out of the two active referents, as in (9). Xiaomei's response starts with the target relative clause presented in a word-by-word moving window format. A sample of the experimental materials is given below:

(8) Context:
       Yidong gongyuli   zhule fangdong yiji  liangge fangke
       one       apartment lived landlord   and two      tenants
       “A landlord and two tenants lived in an apartment.”
       Yiwei zhuhu chaoxingle fangdong
       one    tenant woke up    landlord
       “One of the tenants woke up the landlord.”
       Fangdong ze    chaoxingle lingyiwei zhuhu
        landlord   then woke up    the other tenant
       “The landlord woke up the other tenant.”
Xiaoming: Wo tingshuo qizhong         yiming  zhuhu  hen  gao
                   I     heard     among them   one      tenant  very tall
                   “I heard one of the tenants was very tall.”
                   Nayiwei    zhuhu hen  gao?
                   which.one tenant very tall
                   “Which tenant was very tall?”
(9) Target sentence with a relative clause:
       (i) Standard SR
Xiaomei: Chaoxing fangdong de    zhuhu           hen     gao
                 woke up  landlord   rel  tenant          very    tall
                *V             N             REL Head Noun HN+1 HN+2*
                “The tenant that woke up the landlord was very tall.”
       (ii) Passive SR
Xiaomei: Bei     fangdong chaoxing de     zhuhu           hen      gao
                bei     landlord   woke up rel   tenant           very     tall
                *BEI   N             V            REL Head Noun  HN+1 HN+2*
                “The tenant that was woken up by the landlord was very tall.”
       (iii) Disposal SR
Xiaomei: Ba   fangdong  chaoxing de     zhuhu          hen      gao
                ba   landlord   woke up rel      tenant          very     tall
                *BA  N             V            REL Head Noun  HN+1 HN+2*
                “The tenant that woke the landlord up was very tall.”

Forty-eight additional sets of sentences following a similar format served as fillers. Sixteen of these fillers had relative clauses of various types in them; the remaining 32 fillers did not contain relative clauses. Altogether, 68 sets of contexts and sentences were pseudorandomly presented so that no two experimental trials appeared consecutively. Comprehension questions followed each trial to ensure that participants paid attention in reading the experimental materials. The words used in the relative clauses are provided in the Supplementary Materials.

### Procedure

The experiment followed the standard moving-window self-paced reading design and was conducted using Linger 2.94 (developed by Doug Rohde)^11^. In each trial, participants took their own pace hitting the spacebar to proceed to the next sentence or region. The contexts were presented sentence by sentence, and the target sentences (i.e., Xiaomei's response to Xiaoming's query) were presented word by word. For disposal and passive SRs, *ba*, and *bei* were presented in the same region as the following noun. After the last word, participants were given a true/false comprehension question focusing on the overall content of the context or the target sentence. Feedback was given if the participant's response was incorrect. Participants were instructed to read the sentences at a natural pace in order to answer the comprehension questions correctly. The reading time for each region, the time taken to answer the comprehension questions, and the responses to the comprehension questions were recorded. The whole experiment took an average of 40 min to complete.

## Results

Linear mixed-effects models treating both subjects and items as random effects were fit to both the comprehension accuracy data and the region-by-region reading time data using the lme4 package version 1.1-7 in R (version 3.2.0; Bates et al., 2015). Two contrasts were defined comparing the passive SRs with the standard SRs (passive SR coded as +1, standard SR coded as −1) and comparing the passive SRs with the disposal SRs (passive SR coded as +1, disposal SR coded as −1). The analyses were carried out on log-transformed values of the reading times and residuals were checked to ensure that the normality requirement is met. The package lmerTest (version 2.0-25) in R is used to verify the levels of statistical significance. The *t*-value of 2 is taken to be the threshold for statistical significance at α = 0.05. Question-accuracies were analyzed using generalized linear mixed models with a binomial link function. The dependent measures included comprehension accuracies (binary results), latencies in answering comprehension questions, and region-by-region reading times.

### Comprehension accuracy

**Figure 6:**
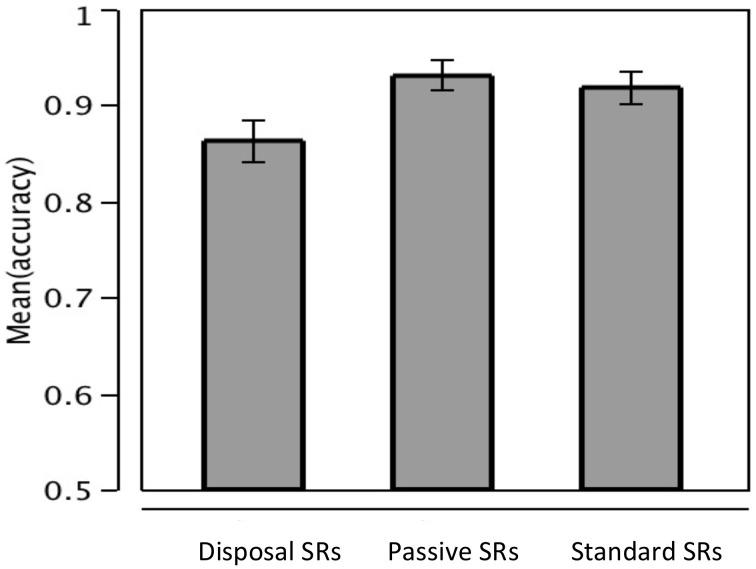
**Comprehension accuracies of disposal SRs, passive SRs, and standard SRs (error bars indicating one standard error)**.

The mean comprehension accuracy for all items was 85% and the mean accuracy for the experimental trials was 90%. The accuracies of each of the three experimental conditions were 93.05% (passive SRs), 91.83% (standard SRs), and 86.28% (disposal SRs). These results are summarized in Figure 6. Statistical results are given in Table 1.

**Table 1:** **Summary of model estimates, standard errors, and the *t* or *z* values for comprehension accuracy and response latency**.

	**Comprehension accuracy**				**Comprehension latency**		
**Contrast**	**Coef**.	***SE***	***z*-values**	***P*(>|*z*|)**	**Coef**.	**SE**	***t*-values**
Intercept	**2.61**	**0.00**	**2105.80**	**<0.001**	**7.67**	**0.06**	**121.44**
Passive_SR-Standard_SR	**0.06**	**0.00**	**51.70**	**<0.001**	0.02	0.03	0.95
Passive_SR-Disposal_SR	**0.71**	**0.00**	**573.80**	**<0.001**	0.01	0.03	0.55

Statistically significant (α = 0.05) effects are highlighted in bold.

In terms of overall comprehension accuracy, passive SRs were comprehended more accurately than both standard SRs and disposal SRs. These results are consistent with the predictions of thematic order effect. Namely, passive SRs, whose thematic order followed the canonical thematic order, were comprehended with greater accuracies than both standard SRs and disposal SRs. No difference was found on the time taken to respond to the comprehension questions.

### Reading times

**Figure 7:**
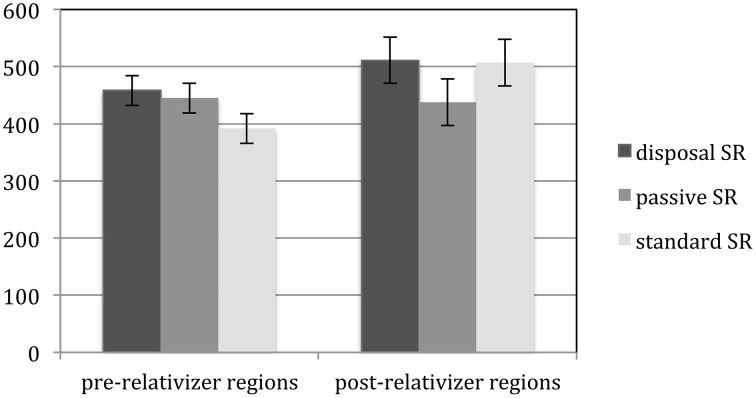
**Average reading times of disposal SRs, passive SRs, and standard SRs in the regions before and after the relativizer (error bars indicating one standard error)**.

Since the regions before and after the relativizers are hypothesized to be reflective of different processing effects, average reading times in the two pre-relativizer regions were compared to those in the post-relativizer regions (from the head noun to two regions after the head noun) across the three conditions. Figure 7 illustrates the results of this analysis. Statistical results are given in Table 2.

**Table 2:** **Summary of model estimates, standard errors, and the *t* values for reading times in the pre-relativizer and post-relativizer regions**.

	**Pre-relativizer regions**			**Post-relativizer regions**		
**Contrast**	**Coef**.	***SE***	***t*-values**	**Coef**.	***SE***	***t*-values**
Intercept	**5.93**	**0.09**	**68.67**	**5.96**	**0.11**	**54.92**
Passive_SR-Standard_SR	**0.11**	**0.02**	**5.08**	**−0.06**	**0.02**	**−2.73**
Passive_SR-Disposal_SR	−0.03	0.02	−1.58	**−0.05**	**0.02**	**−2.37**

Statistically significant (α = 0.05) effects are highlighted in bold.

**Figure 8:**
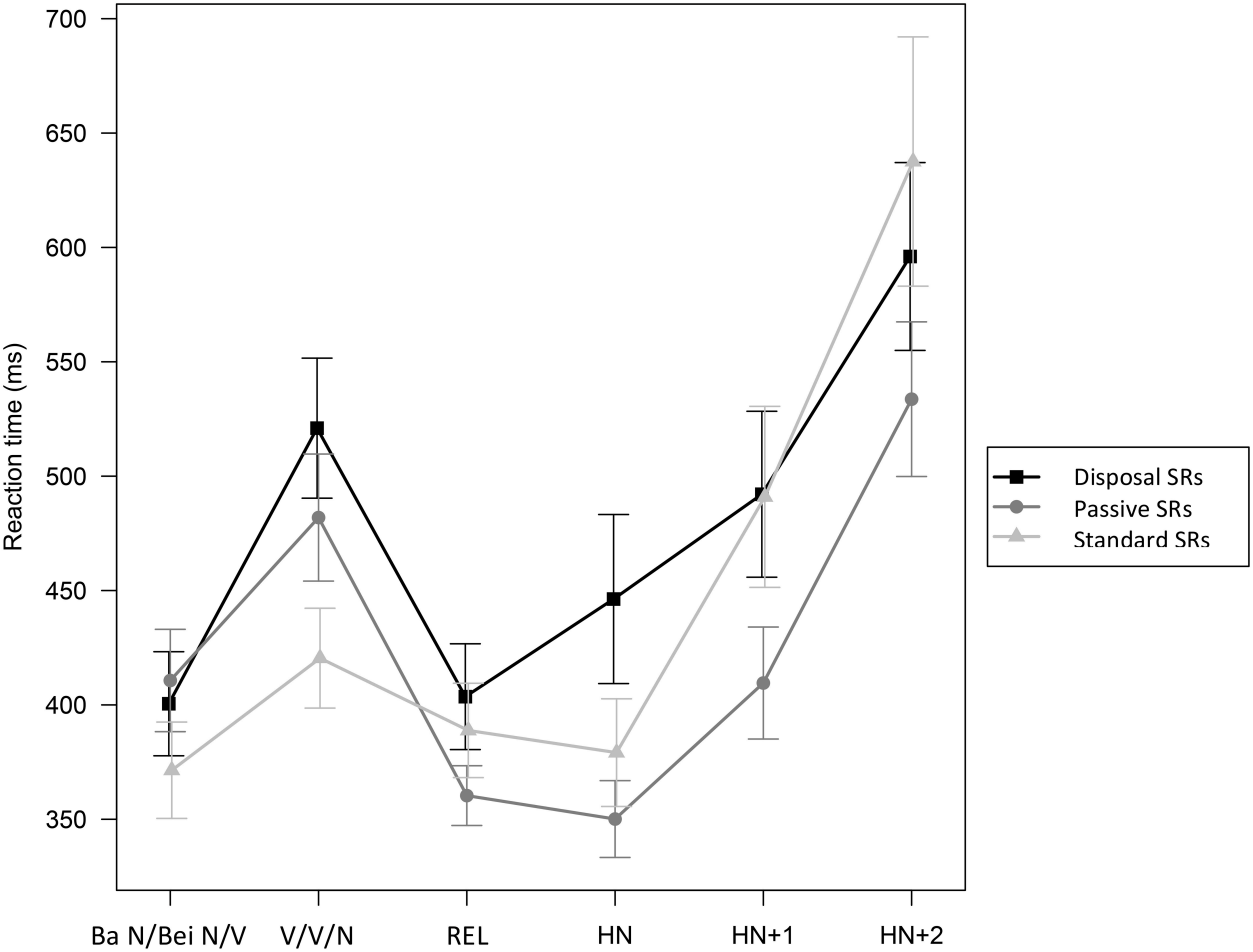
**Reading time of each critical region in the disposal SRs, passive SRs, and standard SRs (error bars indicating one standard error)**. See (9) for region coding.

In the pre-relativizer regions, passive SRs were read longer than standard SRs. In the post-relativizer regions, passive SRs were read faster than both the standard SRs and the disposal SRs. The reading time of each target region, including the two pre-relativizer regions, the relativizer, the head noun, and the two regions after the head noun, is further summarized in Figure 8. Statistical results of the by-region reading time analyses are given in Table 3.

**Table 3:** **Summary of model estimates, standard errors, and the *t* values for reading times in each of the critical regions**.

	**Ba N/Bei N/V**			**V/V/N**			**REL**		
**Contrast**	**Coef**.	***SE***	***t*-values**	**Coef**.	***SE***	***t*-values**	**Coef**.	***SE***	***t*-values**
Intercept	**5.86**	**0.05**	**127.54**	**6.00**	**0.06**	**106.23**	**5.85**	**0.04**	**152.92**
Passive_SR-Standard_SR	**0.09**	**0.03**	**3.47**	**0.13**	**0.03**	**3.89**	−0.02	0.03	−0.91
Passive_SR-Disposal_SR	0.00	0.03	0.07	**−0.07**	**0.03**	**−2.08**	**−0.05**	**0.03**	**−1.97**
	**Head Noun**			**Head Noun** + **1**			**Head Noun** + **2**		
**Contrast**	**Coef**.	***SE***	***t*****-values**	**Coef**.	***SE***	***t*****-values**	**Coef**.	***SE***	***t*****-values**
Intercept	**5.83**	**0.05**	**122.23**	**5.93**	**0.07**	**90.77**	**6.11**	**0.08**	**72.13**
Passive_SR-Standard_SR	−0.04	0.03	−1.11	−0.06	0.04	−1.78	**−0.08**	**0.04**	**−1.96**
Passive_SR-Disposal_SR	**−0.11**	**0.03**	**−3.27**	−0.06	0.04	−1.55	0.01	0.04	0.20

Statistically significant (α = 0.05) effects are highlighted in bold.

Passive SRs were read longer than standard SRs in both regions inside the relative clause (i.e., the pre-relativizer regions), and faster than disposal SRs from the second region in the prenominal clause to the head noun region^12^. In the second region after the head noun, passive SRs were read faster than standard SRs.

To sum up, standard SRs were read with greatest ease in the earlier regions of the relative clauses. In contrast, in the regions following the relativizer, passive SRs were read more quickly than standard SRs and disposal SRs. The easier comprehension of standard SRs in the pre-relativizer regions is consistent both with integration effects (i.e., standard SRs having less complicated dependencies) and with expectation-based constructional frequency effects (i.e., standard SRs being more frequently experienced than passive SRs). The easier comprehension of passive SRs in the post-relativizer regions, on the other hand, is only consistent with the prediction of thematic template mapping.

## General discussion

The present study contrasted the reading patterns of three types of SRs in Chinese: standard SRs, passive SRs, and disposal SRs. Distinctive reading patterns were observed in the regions before and after the relativizer, suggesting the effects of different processing factors being operative. While the current experimental design intends to motivate relative clauses by using referential contexts, it is still unclear whether a relativized gap has indeed been postulated in the pre-relativizer regions given that a relative-clause parse is but one of several possible parses for the pre-relativizer regions. The structurally-ambiguous pre-relativizer regions showed reading patterns consistent with expectation-based theories of sentence comprehension (e.g., the uncertainty-reduction accounts of Hale, 2006 and Chen et al., 2012; see also Jäger et al., 2015), which rely on the probabilities of particular syntactic categories and constituents appearing at particular positions of a sentence. Standard SRs, being the most common prenominal structure of the three, are found to be easier to understand. Besides expectation-based effects, the reading patterns in the pre-relativizer regions are also compatible with integration-based effects, which, as discussed, predict easier processing on structures that involve simpler dependencies. In comparing the three types of SRs, a standard SR involves fewer dependencies and presents a simpler dependency structure.

When the relativizer region is reached, the existence of a relative clause is unambiguously indicated. Consistent with the prediction of the thematic order effect, a passive SR was read faster than the corresponding standard SR and disposal SR given that the thematic order in a passive SR is more frequently experienced than that in a standard SR and that in a disposal SR. All other theoretical factors, by contrast, favor the processing of a standard SR given its structural simplicity and greater constructional frequency. Moreover, this effect of thematic ordering was observed to span across several post-relativizer regions, being attested from the relativizer to the second region after the head noun individually as well as in the sum total. The thematic order effect therefore seems qualitatively different from the gap-filler integration effects, which are usually *localized* to the head noun region.

In previous research on Chinese SR/OR processing, similar asymmetries have been found before and after the relativizer. Recall that, the thematic order of agent-verb-patient found in an OR, which is similar to that in a passive SR, may give a Chinese OR a processing edge over its SR counterpart owing to the thematic order effect. In contrast to an SR, the pre-relativizer regions of a Chinese OR present a word order (i.e., noun-verb) that matches the canonical order in a Chinese sentence and may be read with greater ease than those of a Chinese SR, whose pre-relativizer verb-noun sequence is non-canonical. In previous studies where relative clauses were not structurally disambiguated, greater processing costs were indeed associated with the pre-relativizer regions of an SR—an effect consistent with the prediction of structural probabilities as well as thematic orders (Hsiao and Gibson, 2003; Chen et al., 2008; Qiao et al., 2012). When the relative clauses were structurally disambiguated, however, SRs were processed with greater ease than ORs owing to SRs' greater structural predictability after disambiguating contexts (Jäger et al., 2015)—an effect that is consistent with the prediction of structural probabilities only.

In the post-relativizer regions, an OR disadvantage has been reported for relative clauses modifying the object of an SVO sequence (Lin and Bever, 2006). This effect has been attributed to the reanalysis of a garden-path parse in such structures given that no contextual cues indicated a relative clause parse on the left edge (Lin and Bever, 2011). Most relevant to the current findings, however, in studies that used referential contexts to motivate Chinese relative clauses, an OR advantage consistent with the thematic order effect reported in the current study was obtained (Gibson and Wu, 2013; Lin, 2014).

The thematic order effect on processing Chinese relative clauses is also supported by two offline studies on aphasic patients' processing of Chinese relative clauses: Law and Leung (2000) and Su et al. (2007). Using picture-matching tasks, both studies found better performance on ORs compared to SRs, which was attributed to the fact that Chinese ORs (but not Chinese SRs) match the canonical thematic order. These results are also compatible with the SR advantage of English-speaking aphasic patients (Caplan and Futter, 1986; Grodzinsky, 1986; Hagiwara and Caplan, 1990). An implication of the thematic order effect is that the advantage previously reported for an OR advantage in Mandarin and an SR advantage in English should be re-considered since Mandarin ORs and English SRs, like the passive SRs in the current study, present a canonical thematic order. When comparing SRs and ORs, the advantage for processing Chinese ORs may be due to the ORs presenting canonical thematic orders, but not the SRs.

In the current study, the reading patterns of disposal SRs are contrasted with those of standard SRs and passive SRs. Given their lower constructional frequency and greater number of dependencies involving empty categories, disposal SRs were expected to be the most difficult to process. Indeed, the reading patterns in the present study showed that disposal SRs were the most difficult among the three SRs examined in both the pre- and post-relativizer regions. Given the additional dependencies and lower structural probability associated with passive SRs, it may be expected that they should be equally difficult to process. This result was only obtained for the pre-relativizer regions, where passive SRs were read longer than the standard SRs. In the post-relativizer regions, the reading times of passive SRs were shorter than those of standard SRs and disposal SRs. This can be taken as evidence that the canonical thematic order found in a passive SR induced shorter reading times in its post-relativizer regions. The fact that structural probability effects and thematic template effects have been observed in different regions of a relative clause does not imply that these processes are only operative in different regions of a sentence. Taken together, the results from these different studies suggest that the surprisal-related effect and the thematic template effect are both active and can be independently observed in different regions of a Chinese sentence.

The effect of thematic ordering on sentence comprehension can be understood as a processing heuristic used for efficiently coming up with thematic interpretations for sentences. The sentence processor keeps track of the linear positions of the content words in a sentence in forming thematic interpretations. The dominant thematic order of a language may serve as an “interpretation template,” to which the content words of a sentence are matched. The comprehension of sentences with more complex structures such as relative clauses can be facilitated by matching thematic orders against the dominant thematic templates. Since the dominant thematic template in Chinese is agent-*action*-patient, constructions matching this thematic order (such as ORs and passive SRs) may be comprehended with greater ease. This thematic template effect may also be effective in the comprehension of SRs in English, whose surface thematic order matches the dominant thematic order in the language.

These effects of thematic order are in line with several existing theories of sentence processing. The idea of thematic templates has a similar flavor to Bever's (1970) NVN heuristics—later referred to as “pseudosyntax” in Townsend and Bever (2001). In addition, mapping with thematic templates is also consistent with the “good enough” or “shallow processing” heuristics advanced by Ferreira (2003)^13^. We suggest that in order to arrive at a “good enough” impression of thematic relations, nouns and verbs are matched with the preexisting thematic templates. When the argument order in a sentence follows the dominant thematic template, the thematic roles of the nouns and verbs are easy to identify. Conversely, when the argument order is atypical, it is more difficult to identify thematic relations.

## Conclusion

In conclusion, the reading time data for three sub-types of Chinese SRs reported in the present study supported two processes that are involved in the comprehension of Chinese relative clauses. Before reaching the relativizer, where the structure of the sentence is temporarily ambiguous, expectation-based incremental processing theories such as those of Hale (2001, 2006) and Levy (2008) can account for the processing differences across the three kinds of SRs though the results are also compatible with the integration-based predictions. Starting from the relativizer and the head noun, where the existence of a relative clause is unambiguously indicated, a global effect of thematic ordering was observed.

The critical evidence for the effect of thematic ordering comes from the easier processing of passive SRs, whose thematic role order conforms to the canonical thematic order of Chinese. Despite their more complex structural dependencies and lower constructional frequency compared with standard SRs, passive SRs were nevertheless comprehended with the greatest accuracy and processed with the shortest reading times in the post-relativizer regions. The current study therefore suggests that the comprehension of relative clauses in Chinese is sensitive to both the structural probabilities of constituents as well as the thematic orders involved in the relative clauses. In our effort to understand relative clause comprehension, it is important to take both of these factors into account.

### Conflict of interest statement

The author declares that the research was conducted in the absence of any commercial or financial relationships that could be construed as a potential conflict of interest.

## Footnotes

^1^The classification of these structure-based accounts as working memory accounts is my own. These original structure-based accounts did not necessarily specify the working memory component.

^2^The thematic template theory is of a similar flavor to the NVN strategy of Bever (1970). Whereas, Bever's NVN strategy focuses on the order of syntactic categories in a sentence, the thematic template account focuses on the linear orders of the semantic roles associated with noun phrases in relation to the verb position. What distinguishes the thematic order account from the word order account, therefore, is that the former does not link the semantic arguments to grammatical functions and therefore does not depend on the “structural” positions of the arguments.

^3^In these examples and throughout the paper, rel and asp will be used as abbreviations for “relativizer” and “(perfective) aspect,” respectively.

^4^We adopt a movement/raising analysis for the tree diagrams of subject-extracted and object-extracted relative clauses in Chinese (see Aoun and Li, 2003; Huang et al., 2009).

^5^Alternatively, *bei* has also been analyzed as a preposition taking the NP following it as its oblique object in the long passives and the subject NP as an NP displaced from the object NP in the lower VP (Li, 1990). In this analysis, instead of three dependencies, two dependencies—one between the subject and the lower object and one between the subject gap and the head noun—are involved. While the main verb analysis, which Huang et al. (2009) persuasively argued for, is adopted in the present study, similar predictions about how passive SRs are processed in comparison with standard SRs and disposal SRs can be made when the alternative analysis is adopted.

^6^Like *bei*, the categorical status of *ba* is controversial. In addition to the light verb analysis adopted in the present article, it has also been analyzed as a lexical verb (Hashimoto, 1971), a preposition (Chao, 1968; Li, 1990), and a function word that assigns case (Huang, 1982; Goodall, 1987). In these analyses, the object NP forms a local syntactic constituent with *ba*, through which it is connected with the verb. The dependency between the object NP and the verb is still nested inside the dependency between the subject gap and the head noun.

^7^The integration cost associated with passive SRs may also need to consider the base-generated lowermost trace position, which is linearly closest to the head NP. Even though this short linear dependency may exist between the passivized NP trace and the head noun, it does not preclude the processor from establishing a dependency between the trace NP and the subject gap, which involves a longer linear distance than the dependency in a standard SR.

^8^It is not a simple matter to determine whether a passive SR or a disposal SR is more complex. In terms of the number of dependencies and structural layers, a passive SR is more complex. In terms of the number of different NP identities involved in the dependencies, a disposal SR is more complex. Moreover, while both kinds of SRs exhibit nested dependencies, these dependencies are all associated with the same NP for passive SRs. This factor may make the construction of such dependencies easier to process than the multiple distinct dependencies of a disposal SR. In this sense, then, disposal SRs may be the more complex of the two.

^9^The Sinica Treebank can be found at the following URL: http://turing.iis.sinica.edu.tw/treesearch/. Passive constructions (using *bei*) and disposal constructions (using *ba*) are also less common in Chinese *overall* compared to canonical VO orders. In the Sinica Corpus, *ba* accounted for 14.4% of the words in the corpus and *bei* 14.8% (CKIP online word frequency list http://elearning.ling.sinica.edu.tw/eng_teaching.html, retrieved on September 17, 2012). These overall frequency differences are mirrored in processing differences: Lin (2006, 2008) found that, compared to canonical SVO sentences, disposal sentences and passive sentences showed lower acceptability ratings in naturalness-judgment questionnaires as well as longer reading times in online self-paced reading tasks.

^10^Constructional frequency is but one way to make expectation-based predictions. Alternatively, it is also possible to conduct a sentence completion task to generate word-by-word structural expectations (as has been done in Jäger et al., 2015). The sentence completion task will be particularly useful for distinguishing the processing of passive SRs and disposal SRs. Since the main contrast of interest in the present study is between standard SRs and passive SRs, using corpus counts and constructional frequencies should be sufficient for making the expectation-based predictions. Nonetheless, we leave an actual sentence completion task as an open possibility for generating more fine-grained word-by-word expectation-based predictions.

^11^See http://tedlab.mit.edu/~dr/Linger/ (retrieved on December 9, 2012) for documentation of Linger 2.94.

^12^As a caveat to the advantage of standard SRs observed in the pre-relativizer regions, the disposal SRs and the passive SRs involve an additional function word (i.e., *ba* and *bei*) in the first region, which could induce longer reading times in these regions. The different words in these two pre-relativizer regions also make region-by-region comparisons less straightforward.

^13^In experiments requesting participants to identify the thematic roles of subjects and objects, Ferreira (2003) found that participants made more errors in sentences with atypical thematic orders (e.g., English passive sentences) than sentences with typical thematic orders (e.g., English active sentences). Moreover, this effect was found to be independent of the frequency of the relevant syntactic structures.
