# Supplementary material for: Thematic orders and the comprehension of subject-extracted relative clauses in Mandarin Chinese
Source: Front Psychol. 2015 Sep 11;6:1255. doi: 10.3389/fpsyg.2015.01255 (PMC4566039; doi:10.3389/fpsyg.2015.01255)
Supplement: Supplementary file 1 [file DataSheet1.DOCX]

**Supplementary Material: Nouns and verbs used in the target relative clauses in the experiment.**

| item | N1 | V | N2 (head) |
| --- | --- | --- | --- |
| 1 | 酒吧少爺  ‘bouncer’ | 揍了 ‘beat up’ | 人 ‘guy’ |
| 2 | 重型機車 ‘motorcycle’ | 緊追著 ‘be tailing’ | 轎車 ‘car’ |
| 3 | 高中女學生 ‘high school girl’ | 推了一下‘pushed’ | 婦人 ‘woman’ |
| 4 | 男孩 ‘little boy’ | 打哭 ‘hit to tears’ | 女孩 ‘little girl’ |
| 5 | 休旅車 ‘minivan’ | 撞了 ‘bumped’ | 轎車 ‘car’ |
| 6 | 男孩 ‘boy’ | 請來‘invited here’ | 女孩 ‘girl’ |
| 7 | 男孩 ‘boy’ | 救起‘saved’ | 狗 ‘dog’ |
| 8 | 職業選手 ‘professional runner’ | 推倒‘pushed down’ | 業餘選手 ‘amateur runner’ |
| 9 | 職業殺手 ‘hit man’ | 監視著 ‘be watching’ | 探員 ‘agent’ |
| 10 | 房東 ‘landlord’ | 吵醒 ‘woke up’ | 住戶 ‘tenant’ |
| 11 | 教練 ‘coach’ | 揍傷 ‘beat into bruises’ | 球員 ‘player’ |
| 12 | 指揮家 ‘conductor’ | 惹火 ‘angered’ | 作曲家 ‘composer’ |
| 13 | 女人 ‘woman’ | 抓住 ‘grabbed’ | 男人 ‘guy’ |
| 14 | 機師 ‘pilot’ | 約了 ‘called’ | 空服員 ‘flight attendant’ |
| 15 | 導演 ‘director’ | 大力讚美 ‘praise highly’ | 演員 ‘actor’ |
| 16 | 作家 ‘writer’ | 羞辱 ‘humiliate’ | 記者 ‘reporter’ |
| 17 | 里長 ‘Li governor’ | 帶來 ‘brought’ | 攤販 ‘vendor’ |
| 18 | 包商 ‘contractor’ | 打昏 ‘beat to unconsciousness’ | 歹徒 ‘gangster’ |
| 19 | 校長 ‘principal’ | 告發 ‘denounced’ | 學生 ‘student’ |
| 20 | 台商 ‘Taiwanese merchant’ | 殺死 ‘killed’ | 少年 ‘teenager’ |
